# Colorectal Carcinoma: Local Tumor Staging and Assessment of Lymph Node Metastasis by High-Resolution MR Imaging in Surgical Specimens

**DOI:** 10.1155/2009/659836

**Published:** 2010-01-31

**Authors:** Ichiro Yamada, Norio Yoshino, Akemi Tetsumura, Satoshi Okabe, Masayuki Enomoto, Kenichi Sugihara, Jiro Kumagai, Hitoshi Shibuya

**Affiliations:** ^1^Department of Diagnostic Radiology and Oncology, Graduate School, Tokyo Medical and Dental University, Tokyo 113-8519, Japan; ^2^Department of Oral and Maxillofacial Radiology, Tokyo Medical and Dental University, Tokyo 113-8519, Japan; ^3^Department of Surgery, Tokyo Medical and Dental University, Tokyo 113-8519, Japan; ^4^Department of Pathology, Tokyo Medical and Dental University, Tokyo 113-8519, Japan

## Abstract

*Purpose*. To assess the accuracy of high-resolution MR imaging as a means of evaluating mural invasion and lymph node metastasis by colorectal carcinoma in surgical specimens. *Materials and Methods*. High-resolution T1-weighted and T2-weighted MR images were obtained in 92 surgical specimens containing 96 colorectal carcinomas. *Results*. T2-weighted MR images clearly depicted the normal colorectal wall as consisting of seven layers. In 90 (94%) of the 96 carcinomas the depth of mural invasion depicted by MR imaging correlated well with the histopathologic stage. Nodal signal intensity on T2-weighted images (93%) and nodal border contour (93%) were more accurate than nodal size (89%) as indicators of lymph node metastasis, and MR imaging provided the highest accuracy (94%–96%) when they were combined. *Conclusion*. High-resolution MR imaging is a very accurate method for evaluating both mural invasion and lymph node metastasis by colorectal carcinoma in surgical specimens.

## 1. Introduction

Colorectal carcinoma is one of the most common malignant neoplasms worldwide, and the prognosis is closely correlated with the depth of invasion and the presence of lymph node involvement [1, 2]. Accurate preoperative assessment of these prognostic factors definitely improves the selection of the most appropriate therapy [1, 3]. Computed tomography (CT) and ultrasonography (US) have long been used for staging [3], but depth of cancer invasion and lymph node metastasis cannot be reliably assessed by these methods. Since the assessment of the depth of the cancer in the colorectal wall requires the layers of the colorectal wall to be depicted, endoscopic US has been widely used to assess depth of invasion and lymph node metastasis, but the accuracy of endoscopic US for tumor staging (62%–92%) and nodal metastasis (64%–88%) is still a matter of controversy [3–7]. Thus, the diagnostic methods currently available to evaluate mural invasion and lymph node metastasis are very limited.

Magnetic resonance (MR) imaging also has been used to stage rectal cancer, but previous reports suggest that although conventional MR imaging provides high soft-tissue contrast, it has substantial limitations in regard to T- (primary tumor) staging and N- (regional lymph nodes) staging of rectal cancer because of its limited spatial resolution [8–10]. Thus, high-resolution MR imaging may enable more accurate assessment of depth of invasion and lymph node metastasis by colorectal carcinoma. Previous studies show that high-resolution MR imaging delineates rectal wall layers [11, 12], but its accuracy in T-staging and N-staging of colorectal carcinoma is not known. In vitro study of high-resolution MR imaging is well known to be a useful tool for considering the feasibility of its in vivo application [11, 12]. Thus, the purpose of our study was to assess the accuracy of high-resolution MR imaging as a method of evaluating depth of invasion and lymph node metastasis by colorectal carcinoma in surgical specimens.

## 2. Materials and Methods

### 2.1. Materials

The materials consisted of 92 surgical specimens containing 96 colorectal tumors that had been obtained from 92 consecutive colorectal cancer patients at our institution and that had been histopathologically confirmed to be adenocarcinoma. Sixty-two of the patients were male and 30 were female, and their ages at the time of surgery ranged from 42 to 87 years (mean age: 65 years ± 9 [standard deviation]). The cancers were located in the rectum (*n* = 27), sigmoid colon (*n* = 22), ascending colon (*n* = 21), transverse colon (*n* = 14), cecum (*n* = 9), and descending colon (*n* = 3). The surgical procedure was low anterior resection in 21 patients, abdominoperineal resection in six patients, and segmental resection in 65 patients. Total mesorectal excision was not performed in patients with rectal cancer. No patients in this series received preoperative chemotherapy or radiotherapy.

### 2.2. Imaging Technique

High-resolution MR imaging was performed by using a 1.5 T system with a 25 mT/m maximum gradient capability (Magnetom Vision; Siemens, Erlangen, Germany) and a 4 cm diameter loop coil. All specimens were imaged in vitro after fixation in formalin. Conventional single-section sagittal, coronal, and axial scout images of the colorectal specimen were initially obtained.

High-resolution T1-weighted spin-echo MR images were obtained with a 500/20 (repetition time msec/echo time msec) sequence and with eight signals acquired. High-resolution T2-weighted spin-echo MR images were obtained with a 2000/70 sequence and with four signals acquired. All images were obtained with a 50 × 50-mm field of view, 256 × 256 matrix, and 2 mm slice thickness, yielding a voxel size of 0.076 mm^3^. The interslice gap was 0.5 mm. The bandwidth for the T1-weighted images and T2-weighted images was 65 Hz per pixel and 61 Hz per pixel, respectively, and this was typical for high-resolution MR imaging with the loop coil. Acquisition time for the T1-weighted images and T2-weighted images was 17 minutes 4 seconds and 34 minutes 8 seconds, respectively. The orientation of both the T1- and T2-weighted images was along the longitudinal axis of the resected colon and rectum, so that the entire tumor lesion was imaged. We did not make T2 value measurement or independent NMR spectral measurement.

### 2.3. Image Analysis

The MR images of each lesion were interpreted by two independent radiologists (I.Y., N.Y.), who were blinded to the histopathologic findings. The histopathologic findings were used as the reference standard for analysis of the MR imaging findings. When the radiologists did not fully agree on the findings, the final determination was made by consensus.

*Normal Colorectal Wall.* The high-resolution MR images were reviewed for signal intensity and continuity of each layer of the normal colorectal wall in the 92 surgical specimens. The signal intensity of the layers of the colorectal wall was compared with that of the primary tumor. Thus, we analyzed the signal intensity characteristics of the layers of the normal colorectal wall on the high-resolution MR images.

*Depth of Carcinoma Invasion.* The signal intensity and contour of the primary tumor were analyzed, and the depth of tumor penetration of the colorectal wall was recorded as the deepest layer invaded: mucosa, submucosa, muscularis propria, or subserosa/serosa or adventitia. The MR imaging criteria shown in Table 1 were used by the observers to determine depth of involvement. Both abnormal signal intensity and configuration were used to differentiate between the cancer and the layers of the colorectal wall.

Concerning matching the imaging findings with the pathology findings, we evaluated the deepest invasion area in each tumor lesion on MR images and pathologic sections separately, so as to determine the T-stages of imaging and pathology independently. Thus, the T-stage of MR imaging was matched with the T-stage of pathology in each tumor lesion of the specimens.

*Lymph Node Metastasis.* The MR images of the pericolorectal lymph nodes adjacent to the primary tumor in the specimens were analyzed based on the following three parameters: (a) nodal size, (b) signal intensity, and (c) border contour. (a) Nodal size was measured with electronic calipers on the computer as the maximum diameter of the lymph node in millimeters. (b) The signal intensity of lymph nodes was compared with that of the primary tumor. The signal intensity within the lymph node was classified as uniform or mixed signal, and uniform signal intensity was subclassified as high intensity or iso/low signal intensity. (c) The border contour of a lymph node was classified as “smooth and well-defined” or “irregular and ill-defined.” The “smooth and well-defined” border contour was defined as having an even and regular surface and showing the boundary clearly, whereas the “irregular and ill-defined” border contour was defined as having an uneven surface and showing an unclear boundary. The MR imaging findings of the lymph nodes were compared with histopathologic findings on a node-by-node basis. Spatial correlation between MR images and specimens analysis was achieved by identifying anatomic landmarks (e.g., bowel contour, blood vessels) that were depicted.

### 2.4. Preparation and Examination of the Histopathologic Specimens

After MR imaging the surgical specimen was sectioned longitudinally so that it corresponded to the orientation of the MR images, and the sectioned specimens were embedded in paraffin and cut into 6 *μ*m thick sections with a microtome. The sections were then stained with hematoxylin-eosin (H-E), and a pathologist (J.K.) who was unaware of the MR imaging findings diagnosed the depth of invasion and lymph node metastasis by the carcinoma. 

### 2.5. Statistical Analysis

The sensitivity, specificity, and accuracy of high-resolution MR imaging as a method of assessing the depth of carcinoma invasion and lymph node metastasis were determined by comparison with the histopathologic findings. Depth of invasion according to the MR imaging findings and the histopathologic findings was compared by using the Spearman correlation coefficient. The nodal size of metastatic and nonmetastatic lymph nodes was compared by using Student's *t*-test, and the signal intensity and border contour in the two groups were compared by using the chi-square test. Univariate logistic regression analysis was performed to identify MR imaging findings that could be used to predict lymph node metastasis. Multivariate logistic regression analysis was performed to determine their independent predictive value and to determine what combination of variables (nodal size, signal intensity, and border contour) could best predict lymph node metastasis. The kappa statistic was calculated to determine the interobserver agreement between the two observers for depth of invasion. *P*-values less than .05 were considered indicative of a statistically significant difference. All statistical tests were performed with a statistical software package (StatView, version 5.0; SAS Institute, Cary, NC).

## 3. Results

### 3.1. Signal Intensity of the Layers of the Normal Colorectal Wall

**Figure 1:**
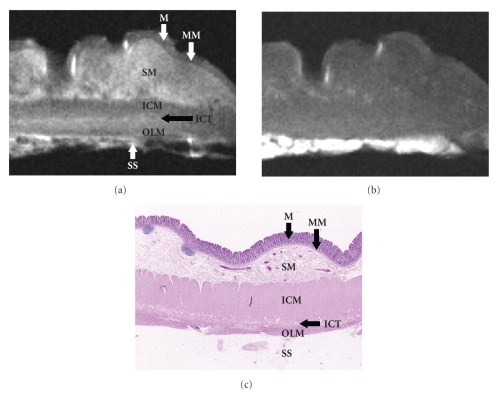
Images of the normal colorectal wall. (a) High-resolution T2-weighted MR image (2000/70) clearly depicts the normal colorectal wall as consisting of seven layers, which correspond well with the histopathologic layers. *M*: mucosa; *MM*: muscularis mucosae; *SM*: submucosa; *ICM*: inner circular muscle; *ICT*: intermuscular connective tissue; *OLM*: outer longitudinal muscle; *SS*: subserosa/serosa or adventitia. (b) High-resolution T1-weighted MR image (500/20) does not depict the detailed structures of the colorectal wall, though subserosal or adventitial fat tissue has high signal intensity. (c) Histopathologic section of the normal colorectal wall shows the mucosa (*M*), muscularis mucosae (*MM*), submucosa (*SM*), muscularis propria (inner circular muscle (*ICM*), intermuscular connective tissue (*ICT*), and outer longitudinal muscle (*OLM*)), and subserosa/serosa or adventitia (*SS*). (Hematoxylin-eosin stain; original magnification: ×3.2.)

High-resolution T2-weighted MR images depicted the mucosa as low signal intensity, and the muscularis mucosae, the deepest layer of the mucosa, as a separate layer that had a lower signal intensity than other parts of the mucosa (Figure 1). The submucosa was high signal intensity, but fat tissue in the submucosa was low signal intensity. High-resolution T2-weighted MR images separated the muscularis propria into three layers. The inner circular muscle layer and outer longitudinal muscle layer were seen as discrete low-signal-intensity structures separated by a thin, high-signal-intensity band that correlated with the loose connective tissue histopathologically. The subserosa/serosa or adventitia appeared as high signal intensity, but fat tissue in the subserosa or adventitia was visualized as low signal intensity.

Thus, the high-resolution T2-weighted MR images clearly depicted the normal colorectal wall as consisting of the following seven layers that correlated well with the layers of the colorectal wall histopathologically: mucosa (low signal intensity), muscularis mucosae (low signal intensity), submucosa (high signal intensity), inner circular muscle layer (low signal intensity), intermuscular connective tissue (high signal intensity), outer longitudinal muscle layer (low signal intensity), and subserosa/serosa or adventitia (high signal intensity).

The high-resolution T1-weighted MR images depicted the mucosa, muscularis mucosae, submucosa, muscularis propria, and subserosa/serosa or adventitia in the colorectal wall as having similar low signal intensity, and the fat tissue in the submucosa and subserosa or adventitia as high signal intensity (Figure 1).

On the high-resolution T2-weighted MR images, the colorectal wall appeared as seven layers in 44 (48%) of the 92 specimens. In 46 (50%) specimens, however, the colorectal wall appeared as six layers because the muscularis mucosae was not separated from the mucosa. In the remaining two (2%) specimens, only four layers were observed because the muscularis mucosae was not separated from the mucosa and the muscularis propria appeared as a single low-signal-intensity zone.

### 3.2. Evaluation of the Depth of Carcinoma Invasion

**Figure 2:**
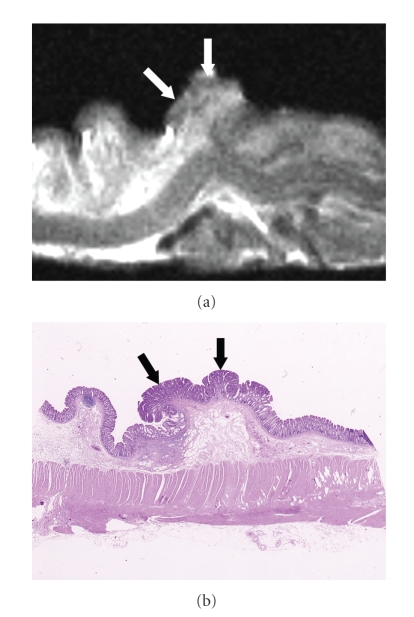
Colorectal carcinoma confined within the mucosa. (a) High-resolution T2-weighted MR image (2000/70) shows an irregular thickening (arrows) in the mucosa, and the submucosa of high signal intensity appears to be intact. (b) Corresponding histopathologic section shows carcinoma confined within the mucosa (arrows) as well as intact submucosa. (Hematoxylin-eosin stain; original magnification: ×2.5.)

**Figure 3:**
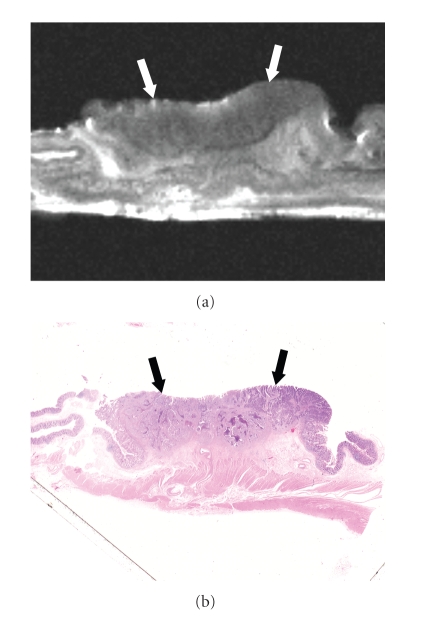
Colorectal carcinoma invading the submucosa. (a) High-resolution T2-weighted MR image (2000/70) shows that an irregularly-shaped tumor (arrows) contrasts with the high-signal-intensity submucosa. (b) Corresponding histopathologic section shows carcinoma invading the submucosa (arrows). (Hematoxylin-eosin stain; original magnification: ×1.6.)

**Figure 4:**
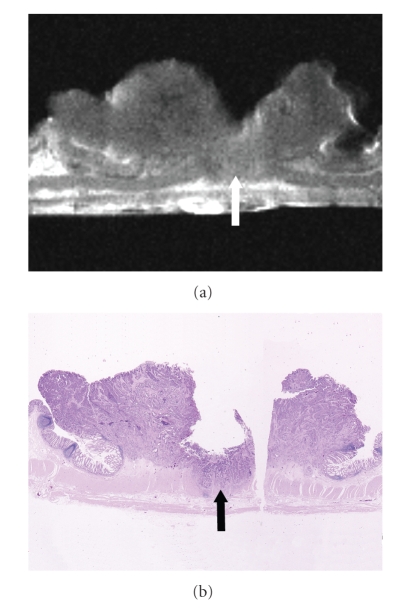
Colorectal carcinoma involving the muscularis propria. (a) High-resolution T2-weighted MR image (2000/70) shows that an irregularly-shaped tumor partially replaces the muscularis propria layer (arrow), but that it does not penetrate through the muscularis propria layer. There is a deep ulceration in the central part of the tumor. (b) Corresponding histopathologic section shows carcinoma involving the muscularis propria (arrow) which manifests a deep ulceration in the central part. (Hematoxylin-eosin stain; original magnification: ×1.8.)

At histopathologic examination, the 96 colorectal carcinomas in this series consisted of 19 carcinomas confined to the mucosa, 15 that had invaded the submucosa, 15 that had infiltrated the muscularis propria, and 47 that had extended into the subserosa/serosa or adventitia (Table 2). The signal intensity of the colorectal carcinomas varied with the histopathologic components of the tumor (Figures 2–4). The epithelial component of the primary tumor was low to intermediate signal intensity on the high-resolution T2-weighted images and low signal intensity on the high-resolution T1-weighted images.

The depth of carcinoma invasion of the colorectal wall was clearly demonstrated by high-resolution T2-weighted MR imaging. On the high-resolution T2-weighted MR images carcinomas confined to the mucosa were visualized as a discrete low-signal-intensity thickening in the mucosal layer (Figure 2), but the submucosa appeared intact. Carcinomas that had invaded the submucosa were demonstrated as irregular low-signal-intensity mass lesions that contrasted with the high-signal-intensity submucosa on high-resolution T2-weighted MR images (Figure 3). Carcinomas involving the muscularis propria appeared as tumor lesions that had partially replaced the muscularis propria layer (Figure 4). Carcinomas extending into the subserosa/serosa or adventitia were depicted as tumor lesions that had completely disrupted the muscularis propria layer and invaded the subserosa/serosa or adventitia.

As shown in Table 2, in 90 (94%) of the 96 colorectal carcinomas the results for depth of mural invasion obtained by high-resolution MR imaging were the same as the results for depth of invasion determined histopathologically. The stage of invasion determined by high-resolution MR imaging, however, was higher than determined histopathologically in five (5%) carcinomas, and lower in one (1%) carcinoma. MR imaging overestimated two mucosal carcinomas as having invaded the submucosa, one submucosal carcinoma as having invaded the muscularis propria, and two muscularis propria carcinomas as having invaded the subserosa/serosa or adventitia. MR imaging underestimated one carcinoma that had involved the subserosa/serosa or adventitia as only having invaded to the muscularis propria.

Table 3 shows the accuracy of each of the diagnostic criteria employed in the evaluation of depth of invasion on high-resolution MR images (Table 1). MR imaging enabled correct diagnosis of depth of invasion in all 96 colorectal carcinomas that had invaded the mucosa, and thus both its sensitivity and accuracy for diagnosis of mucosal invasion were 100%. Its specificity was not determined. MR imaging allowed correct diagnosis of all 77 lesions that had invaded the submucosa, but submucosal invasion was misdiagnosed in two lesions in which there was no submucosal invasion. Thus, the sensitivity, specificity, and accuracy of diagnosis of submucosal invasion were 100%, 89%, and 98%, respectively. MR imaging allowed correct diagnosis of depth of invasion in all 62 lesions that had invaded the muscularis propria, but muscularis propria invasion was misdiagnosed in one lesion in which there was no muscularis propria invasion. Thus, the sensitivity, specificity, and accuracy of diagnosis of muscularis propria invasion were 100%, 97%, and 99%, respectively. MR imaging enabled correct diagnosis of depth of invasion in 46 of the 47 lesions that had invaded subserosa/serosa or adventitia, but the other lesion was misdiagnosed as having invaded the muscularis propria, and two lesions with no invasion of the subserosa/serosa or adventitia were misdiagnosed as having invaded the subserosa/serosa or adventitia. Thus, the sensitivity, specificity, and accuracy for diagnosis of subserosa/serosa or adventitia invasion were 98%, 96%, and 97%, respectively.

A Spearman coefficient (*r* value) of 0.971 was obtained for the correlation between the diagnoses of depth of invasion by MR imaging and histopathologically (*P* < .0001), and thus the correspondence was excellent (slope = 0.999, intercept value = −0.040). In eight (8%) of the 96 carcinomas the observers did not agree on depth of invasion, and the diagnosis was made by consensus. The kappa value of 0.876 was obtained between the two observers, and thus the interobserver agreement for depth of invasion was excellent.

### 3.3. Evaluation of Lymph Node Metastasis

Since high-resolution MR imaging depicted 82 lymph nodes in the 92 colorectal specimens that were examined histopathologically, we compared the high-resolution MR imaging findings and histopathologic findings in the 82 lymph nodes. The 82 lymph nodes confirmed by pathology were in 33 (36%) of the 92 surgical specimens. The number of lymph nodes in these specimens ranged from one to eight, and the median number of lymph nodes was two (the mean number: 2.5). Other lymph nodes also were separately harvested at surgery from the 92 patients, but these lymph nodes were not analyzed because they were not imaged with high-resolution MR imaging. Histopathologic examination revealed metastasis in 32 (39%) of the 82 lymph nodes and no metastasis in the other 50 (61%) lymph nodes.

**Figure 5:**
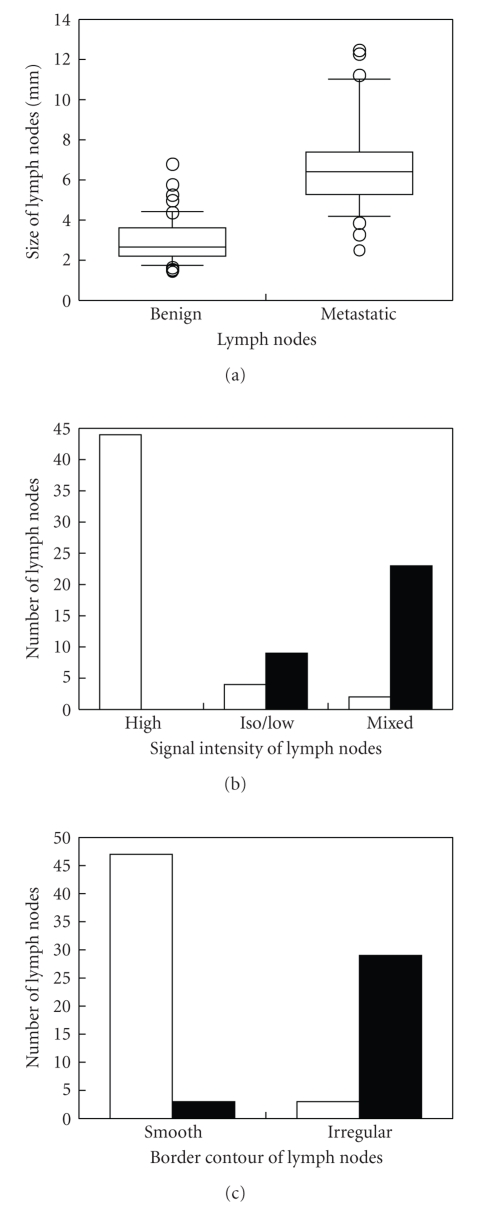
The size, signal intensity, and border contour of lymph nodes in colorectal carcinomas on high-resolution MR images. (a) Box plot shows the diameter (measured in millimeters) of benign and metastatic lymph nodes on high-resolution MR images. The horizontal line within the box is the median value (50th percentile), the boundaries of the box represent 25th and 75th percentiles, and whiskers show 10th and 90th percentiles. Values above the 90th and below the 10th percentiles are plotted as data points (circles). (b) Bar chart shows the number of benign (white bars) and metastatic (black bars) lymph nodes on high-resolution MR images, according to signal intensity on T2-weighted MR images. (c) Bar chart shows the number of benign (white bars) and metastatic (black bars) lymph nodes on high-resolution MR images, according to border contour.

The evaluation of nodal size showed that the metastatic lymph nodes ranged from 2.6 mm to 12.6 mm in size and that the benign lymph nodes ranged from 1.5 mm to 6.9 mm in size (Figure 5(a)). The metastatic lymph nodes (6.9 mm ± 2.5) were statistically significantly larger than the benign lymph nodes (3.0 mm ± 1.1) (*P* < .0001), but, as shown in Figure 5(a), there was a considerable overlap between the sizes of the metastatic lymph nodes and the benign lymph nodes.

**Figure 6:**
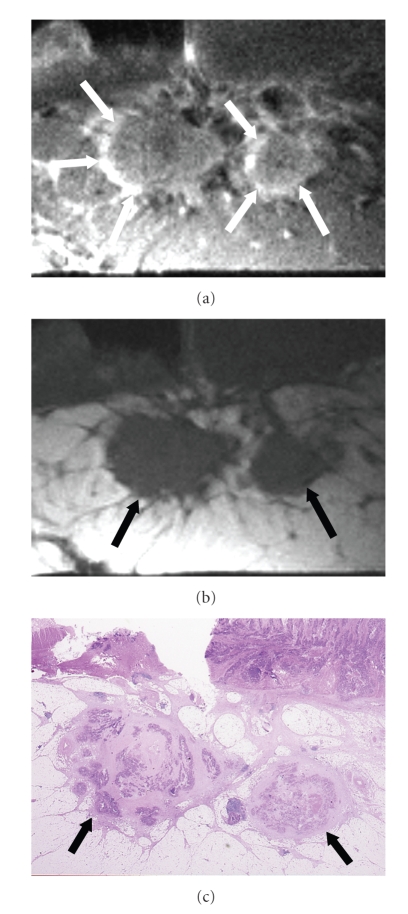
Metastatic lymph nodes showing mixed signal intensity on high-resolution T2-weighted images and irregular border contour. (a) High-resolution T2-weighted MR image (2000/70) shows lymph nodes (arrows) having mixed signal intensity and irregular border contour in the subserosal fat. (b) High-resolution T1-weighted MR image (500/20) shows lymph nodes (arrows) having irregular border contour. (c) Corresponding histopathologic section shows metastatic lymph nodes (arrows) in the subserosa. (Hematoxylin-eosin stain; original magnification: ×1.3.)

**Figure 7:**
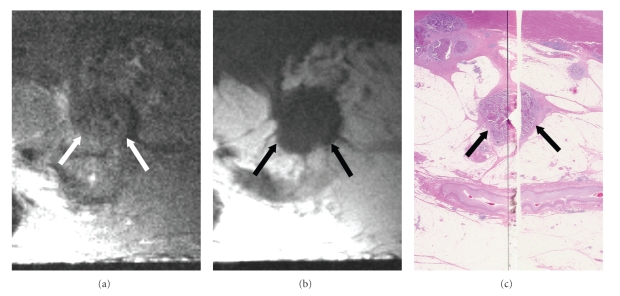
Metastatic lymph nodes showing low signal intensity on high-resolution T2-weighted images and irregular border contour. (a) High-resolution T2-weighted MR image (2000/70) shows lymph nodes (arrows) having low signal intensity and irregular border contour in the subserosal fat. (b) High-resolution T1-weighted MR image (500/20) shows lymph nodes (arrows) having irregular border contour. (c) Corresponding histopathologic section shows metastatic lymph nodes (arrows) in the subserosa. (Hematoxylin-eosin stain; original magnification: ×1.16.)

**Figure 8:**
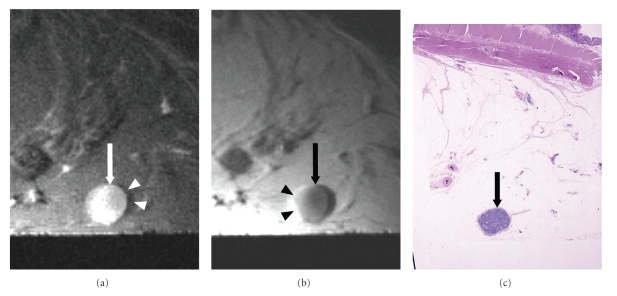
Benign lymph nodes showing high signal intensity on high-resolution T2-weighted images and smooth border contour. (a) High-resolution T2-weighted MR image (2000/70) shows lymph nodes (arrow) having high signal intensity and smooth border contour in the subserosal fat. The chemical shift artifact (arrowheads) is noted. (b) High-resolution T1-weighted MR image (500/20) shows lymph nodes (arrow) having smooth border contour. The chemical shift artifact (arrowheads) is noted. (c) Corresponding histopathologic section shows benign lymph nodes (arrow) in the subserosa. (Hematoxylin-eosin stain; original magnification: ×1.25.)

The evaluation of signal intensity revealed metastasis in 23 (92%) of the 25 lymph nodes showing mixed signal intensity on high-resolution T2-weighted images and no metastasis in the other two (8%) lymph nodes (Figures 5(b) & 6). Metastasis was found in nine (69%) of the 13 lymph nodes depicted as iso/low signal intensity and no metastasis was found in the other four (31%) (Figure 7). None (0%) of the 44 lymph nodes visualized as high signal intensity contained metastases, and all of them (100%) were metastasis-free (Figure 8). There was a statistically significant difference in the signal intensity between the metastatic and benign lymph nodes (*P* < .0001).

The evaluation of border contour showed that 29 (91%) of the 32 lymph nodes with an irregular, ill-defined border contour were metastatic, and the other three (9%) were metastasis-free (Figures 5(c), 6 & 7). Three (6%) of the 50 lymph nodes with a smooth, well-defined border contour contained a metastasis, and the other 47 (94%) were metastasis-free (Figure 8). There was a statistically significant difference between the border contours of the metastatic and benign lymph nodes (*P* < .0001).

Table 4 shows the diagnostic accuracy of high-resolution MR imaging as a method of evaluating lymph node metastasis by colorectal carcinoma. The accuracy of the nodal size criteria for lymph node metastasis varied with the cutoff value, and the cutoff value of ≥4 mm resulted in greater accuracy (89% [73/82]) than the other cutoff values. Mixed signal intensity or iso/low signal intensity resulted in greater accuracy (93% [76/82]) as a criterion than mixed signal intensity alone, and an irregular, ill-defined border contour resulted in high accuracy (93% [76/82]) as a criterion for lymph node metastasis. High-resolution MR imaging provided greater accuracy (94% [77/82]-96% [79/82]) for evaluating lymph node metastasis when the combination of nodal size, signal intensity, and border contour was used.

Both univariate and multivariate logistic regression analyses revealed nodal size, signal intensity, and border contour as significant parameters for predicting lymph node metastasis. The multivariate logistic regression analysis demonstrated the model which could best predict lymph node metastasis from nodal size, signal intensity, and border contour, with the following equation for the logit:

(1) $$\begin{array}{cc} \text{logit} & =-9.509+1.247\times \left(\text{size}\right)+3.561\times \left(\text{signal}\right)\\ & \;+4.463\times \left(\text{border}\right), \end{array}$$

where size = nodal size (mm), signal = 0 (for high or iso/low signal intensity) or 1 (for mixed signal intensity), and border = 0 (for smooth border) or 1 (for irregular border). The probability *P* of lymph node metastasis can be calculated with the following formula:

(2) $$P=\frac{\exp\;\;\left(\text{logit}\right)}{1+\exp\;\;\left(\text{logit}\right)}.$$

## 4. Discussion

Our findings demonstrated that high-resolution T2-weighted MR images clearly depicted the normal colorectal wall as consisting of seven layers that corresponded well with the actual layers of the colorectal wall observed histopathologically. High-resolution MR imaging provides much higher soft-tissue contrast than CT or US, and there are none of the artifactual interface echoes in the colorectal wall that occur with US. Previous studies have described the colorectal wall as consisting of three to six layers on T2-weighted MR images [9, 12, 13], whereas our results demonstrated that high-resolution T2-weighted MR imaging clearly depicts the actual layers of the colorectal wall observed histopathologically.

Our findings showed that high-resolution MR imaging was able to correctly depict the depth of invasion of the colorectal wall in 90 (94%) of the 96 colorectal carcinomas studied. Although the assessments of depth of invasion by MR imaging resulted in overestimation in five (5%) of the other carcinomas and underestimation in other one (1%), the ranges of its sensitivity, specificity, and accuracy as a method of assessing depth of invasion of the colorectal wall were 98%–100%, 89%–97%, and 97%–100%, respectively. Thus, high-resolution MR imaging was found to be a highly accurate method for evaluating depth of invasion by colorectal carcinoma. Its high accuracy appears to be attributable to the combination of high soft-tissue contrast and high spatial resolution that high-resolution MR imaging provides [8–20]. Its diagnostic accuracy, however, should be cautiously compared with previous studies that were performed in vivo, because the high-resolution MR imaging data in our study were obtained from fixed surgical specimens.

Our findings also demonstrated a high degree of accuracy of high-resolution MR imaging for evaluating lymph node metastasis by colorectal carcinoma. Nodal signal intensity on high-resolution T2-weighted images (93%) and nodal border contour (93%) provided greater accuracy than nodal size (89%). MR imaging yielded the greatest accuracy (94%–96%) for evaluating lymph node metastasis when nodal size, signal intensity and border contour were combined.

Previous reports indicate that lymph node evaluation in rectal cancer is challenging for every imaging technique, because lymph node size alone is not a reliable diagnostic criterion for metastatic involvement [8, 21]. Conventional MR imaging also has substantial limitations in regard to the N-staging of rectal cancer [8–10]. Bipat et al. [8] reported that conventional MR imaging for N-staging of rectal cancer had a sensitivity of 66% (54%–76% [95% confidence intervals]) and a specificity of 76% (59%–87%). Recent papers show that lymph node evaluation in rectal cancer patients is improved by examining the morphologic characteristics of the lymph nodes on MR images [22, 23], and MR imaging with ultrasmall particles of iron oxide (USPIO) has shown promising results for assessing lymph node metastasis in rectal cancer [24].

A limitation of our study is that the specimens were imaged after fixation in formalin. However, since previous reports have shown no substantial effect of formalin fixation, on the signal intensity and soft-tissue contrast of T2-weighted images in the colorectal wall, gastric wall, and esophageal wall [11, 25, 26], the findings in our study may be applicable to high-resolution MR imaging of in vivo specimens as well as formalin-fixed specimens.

Another limitation of our study is that we analyzed only the pericolorectal lymph nodes adjacent to the primary tumor in the specimens, and thus more proximal lymph nodes were not analyzed, even when separately harvested at surgery. However, this procedure enabled strict node-by-node correlations to be made with the histopathologic findings, and in our study it was possible to strictly determine whether individual lymph nodes on high-resolution MR images contained metastases. Studies using a patient-by-patient analysis alone might be insufficient to clarify imaging findings of individual lymph nodes containing metastases [22–24]. Therefore, our procedure was helpful for assessing the accuracy of the high-resolution MR imaging findings as a means of evaluating lymph node metastasis by colorectal carcinoma.

Finally, since our results were obtained by imaging surgical specimens using a dedicated coil and long acquisition times, the results cannot be directly extrapolated to clinical practice. There are many technical issues associated with performing the high-resolution technique in vivo, including bowel peristalsis, patient motion, bowel collapse, residual fecal material and gas, and distance from the coil to the lesion. However, high-resolution MR imaging in vivo may become possible by using an endoluminal coil technique or phased-array coil technique and with the development of faster MR imaging techniques. Higher field strength (3.0 T) also may enable high-resolution MR imaging in reduced acquisition times.

In conclusion, our study has demonstrated that high-resolution MR imaging clearly depicts the internal architecture of the colorectal wall in surgical specimens and is a highly accurate diagnostic method for evaluating depth of invasion and lymph node metastasis by colorectal carcinoma. Thus, high-resolution MR imaging may enable accurate preoperative local tumor staging and lymph node assessment of colorectal carcinomas.

## Figures and Tables

**Table 1:** MR imaging criteria used to determine the depth of invasion in colorectal carcinoma.

Depth of invasion	MR imaging criteria
Mucosa (Tis)	Thickening in the mucosal layer
Submucosa (T1)	Mass in the submucosal layer
Muscularis propria (T2)	Mass extending into the muscle layer
	Abnormal signal intensity in the thickened muscle layer
Subserosa/serosa or adventitia (T3 and T4)	Mass extending through the muscle layer into the subserosa or adventitia

Note. Letters in parentheses indicate the corresponding tumor stage according to the International Union against Cancer Tumor-Node-Metastasis classification [2]. Tis: carcinoma in situ.

**Table 2:** Comparison of high-resolution MR imaging and histopathologic findings for evaluating the depth of invasion in colorectal carcinoma.

	Histopathologic findings			
MR imaging findings	Mucosa(*n* = 19)	Submucosa (*n* = 15)	Muscularis propria (*n* = 15)	Subserosa/serosa or adventitia (*n* = 47)
Mucosa	17	0	0	0
Submucosa	2	14	0	0
Muscularis propria	0	1	13	1
Subserosa/serosa or adventitia	0	0	2	46

Note. Numbers are numbers of lesions among 96 carcinomas in 92 patients.

**Table 3:** Diagnostic accuracy of high-resolution MR imaging for evaluating the depth of invasion in colorectal carcinoma.

Depth of invasion	Sensitivity	Specificity	Accuracy
Mucosa	96/96 (100)	0/0 (NA)	96/96 (100)
Submucosa	77/77 (100)	17/19 (89)	94/96 (98)
Muscularis propria	62/62 (100)	33/34 (97)	95/96 (99)
Subserosa/serosa or adventitia	46/47 (98)	47/49 (96)	93/96 (97)

Note. Data represent the diagnostic accuracy for invasion of each layer of the colorectal wall in 96 carcinomas. Values in parentheses are percentages. NA: not applicable.

**Table 4:** Diagnostic accuracy of high-resolution MR imaging for evaluating lymph node metastasis in colorectal carcinoma.

Criterion for lymph node metastasis	Sensitivity	Specificity	Accuracy
Nodal size			
≥8 mm	7/32 (22)	50/50 (100)	57/82 (70)
≥7 mm	11/32 (34)	50/50 (100)	61/82 (74)
≥6 mm	21/32 (66)	49/50 (98)	70/82 (85)
≥5 mm	26/32 (81)	46/50 (92)	72/82 (88)
≥4 mm	29/32 (91)	44/50 (88)	73/82 (89)
≥3 mm	31/32 (97)	29/50 (58)	60/82 (73)
Signal intensity (SI)			
Mixed	23/32 (72)	48/50 (96)	71/82 (87)
Mixed or iso/low	32/32 (100)	44/50 (88)	76/82 (93)
Border contour			
Irregular	29/32 (91)	47/50 (94)	76/82 (93)
Mixed or iso/low SI and irregular border			
	29/32 (91)	50/50 (100)	79/82 (96)
Nodal size ≥4 mm and (mixed or iso/low SI or irregular border)			
	29/32 (91)	48/50 (96)	77/82 (94)

Note. Values in parentheses are percentages.
